# Hypo-Fractionated Stereotactic Radiosurgery for the Management of Brain Metastases

**DOI:** 10.3390/cancers17183026

**Published:** 2025-09-16

**Authors:** Stylianos Pikis, Georgios Mantziaris, Kimball Sheehan, Darrah Sheehan, Jason P. Sheehan

**Affiliations:** 1 Stereotactic Radiosurgery and Radiation Oncology Unit, Mediterraneo Hospital, 16675 Glyfada, Greece; steliospikis78@gmail.com; 2 Neurosurgery, EIMC Clinic, 2023 Nicosia, Cyprus; 3 Department of Neurological Surgery, University of Virginia Health System, Charlottesville, VA 22903, USA; urf4wf@uvahealth.org (G.M.); kimballsheehan@gmail.com (K.S.); darrahsheehan@gmail.com (D.S.)

**Keywords:** stereotactic radiosurgery, hypo-fractionated, brain metastases, radiobiology

## Abstract

The increasing prevalence of brain metastases in cancer patients due to longer life expectancy and improvements in neuroimaging highlights the need for effective local treatments. Despite advancements in systemic targeted therapies, their low blood–brain barrier (BBB) penetrance limits their intracranial efficacy. Stereotactic radiosurgery (SRS) has largely supplanted whole-brain radiation therapy (WBRT) for patients with up to 10 brain lesions due to superior neurocognitive outcomes and high local control. While single-fraction SRS provides low radiation toxicity with smaller lesions, high-volume metastases necessitate doses above tolerance limits to achieve comparable local control. As tumor volume increases, the number of tumor cells also increases, requiring higher doses of radiation than the maximum tolerated doses reported in the RTOG 9005 study to achieve tumor control. Hypo-fractionated SRS (HySRS) permits the delivery of high radiation doses over 2–5 fractions, thus mitigating the risk of radiation toxicity while maintaining high local control. This review presents the available evidence and ongoing clinical trials on HySRS for the management of brain metastases.

## Simple Summary

Improved brain imaging and longer life expectancy of cancer patients have led to an increased prevalence of brain metastases. While new systemic treatments can provide high extracranial disease control, their low penetrance into the brain means that focal treatment, such as stereotactic radiosurgery, is still necessary to maintain intracranial disease control. Recent advancements in image guidance and radiosurgical techniques have allowed for delivering stereotactic radiosurgery in multiple fractions (hypo-fractionated). This technique maintains high tumor control while minimizing radiation toxicity to nearby brain structures. This review summarizes the current literature and provides information on ongoing clinical trials on hypo-fractionated stereotactic radiosurgery for brain metastases.

## 1. Introduction

Brain metastases occur in up to 10–40% of all cancer patients, and their prevalence is increasing due to longer life expectancies afforded by new systemic therapies and wider use of advanced brain MRI [1]. Advancements in systemic therapies have also resulted in improved survival of patients with brain metastases, underscoring the significance of providing effective local treatment while minimizing treatment-related toxicity.

Stereotactic radiosurgery (SRS) has emerged as the standard of care for selected patients with up to 10 lesions, mainly due to comparable local control and superior neurocognitive outcomes compared to whole-brain radiation therapy (WBRT) [2,3]. Though single-fraction SRS achieves high rates of local control for small tumors, the Radiation Therapy Oncology Group (RTOG) trial 9005 demonstrated that the ability to safely deliver high doses of radiation in a single fraction is limited when the lesion diameter exceeds 2 cm. In that dose escalation study of single-fraction SRS for previously irradiated solitary brain metastasis and gliomas, the maximum tolerated dose established was 24 Gy, 18 Gy, and 15 Gy for lesions < 2 cm, 2–3 cm, and 3–4 cm in maximum diameter, respectively [4]. However, as tumor volume increases, the number of tumor cells also increases, requiring higher doses of radiation than the maximum tolerated doses reported in the RTOG 9005 study to achieve tumor control. In the Hypo-fractionated Treatment Effects in the Clinic (HyTEC) study, inadequate tumor control of lesions >2 cm with the dose constraints established by the RTOG 9005 trial were noted, suggesting that fractionated treatment strategies should be employed in this setting [5].

Recent advancements in image-guided technology have allowed for the delivery of SRS in 2–5 fractions [i.e., hypo-fractionated SRS (HySRS)] using masks instead of the traditional rigid frame. HySRS permits the delivery of high radiation doses while allowing for normal brain tissue repair between fractions, thus mitigating the risk of radiation toxicity while maintaining high tumor.

In this article, we review the current evidence and ongoing clinical trials on HySRS for the management of brain metastases.

## 2. Hypo-Fractionated Stereotactic Radiosurgery for Brain Metastases

### 2.1. Radiobiology of Stereotactic Radiosurgery and Hypo-Fractionate Radiosurgery

The effect of fractionated radiation therapy on tumors is determined by five radiobiological factors known as the 5 Rs [6,7]:

1. Repair of radiation-induced sub-lethal single- and double-stranded DNA damage.
2. Redistribution of cells in the more radiosensitive G1 and early S phases of the cell cycle.
3. Re-oxygenation of hypoxic cells, rendering them more radiosensitive.
4. Repopulation of cancer cells between fractions, which may lead to failure of radiotherapy.
5. Radiosensitivity.

The linear quadratic (LQ) model describes the relationship between radiation dose per fraction and tumor cell survival. According to the LQ model, single- and double-hit kill mechanisms plotted as linear and quadratic terms, respectively, are responsible for cell survival. At low radiation doses, the surviving cell fraction is linearly proportional to the radiation dose administered and decreases more rapidly with moderate doses. The α/β ratio represents the dose at which the linear (α) and the quadratic (β) components equally contribute to cell kill. Slowly dividing cells (brain) have low α/β ratio and respond late to radiation. Rapidly dividing cells (malignant tumors) are more sensitive to lower doses of radiation, have a high α/β ratio, and respond early to radiation. Though the LQ model has been well studied for multi-fraction, low-dose radiotherapy, its validity when using ablative radiation doses (>8 Gy) during SRS remains controversial. Advocates of the LQ model suggest that the radiobiologic principles valid for conventional radiotherapy are sufficient to explain the efficacy of SRS [8]. On the other hand, opponents of the LQ model propose that in addition to direct cell death from DNA damage, ablative radiation doses induce different radiobiologic mechanisms such as damage of the vascular endothelium and enhanced anti-tumor immunity leading to indirect tumor-cell death [9].

The LQ model also allows for the calculation of the biologically effective dose (BED) for different total doses (D) and dose per fraction (d):

BED α/β = D [1 + (d/α/β)]

As dose per fraction increases, the BED of low α/β ratios tissues will increase much more rapidly than the BED of tissues with high α/β ratios, suggesting that fractionated regimens will result in less toxicity to normal tissue than single-fraction SRS at the same total dose. Therefore, the difference in the α/β ratio between tumor and normal tissue cells can be exploited to widen the therapeutic window and improve the therapeutic ratio.

Single-fraction SRS enables the delivery of a high radiation dose with sub-millimeter accuracy and a steep dose fall-off outside the target, minimizing but not eliminating the volume of normal tissue exposed to radiation. In the HyTEC study, single-fraction SRS for brain metastases was associated with a 10%, 15%, and 20% risk of symptomatic adverse radiation effects with 12-Gy volumes of 5 cm^3^, 10 cm^3^, and >15 cm^3^, respectively [10]. Mayo et al. reported a low complication risk of brainstem radiation-induced injury and radiation-induced optic neuritis for single-fraction SRS with brainstem and optic nerve Dmax < 12.5 Gy and ≤10 Gy, respectively [11,12]. Therefore, the ability to deliver high doses of radiation in a single fraction is limited with large volume brain metastases and with those lesions adjacent to radiation-sensitive structures, such as the optic nerves and the brainstem. In this setting, HySRS may be considered as an alternative to single-fraction SRS to maintain high tumor control while mitigating the risk of complications. HySRS allows for inter-fractional re-oxygenation and redistribution of tumor cells in “radiosensitive” stages of the cell cycle, thereby improving tumor cell kill, while at the same time it allows for repair of normal cells, minimizing toxicity.

### 2.2. Hypo-Fractionated SRS for Intact Brain Metastases

**Figure 1:**
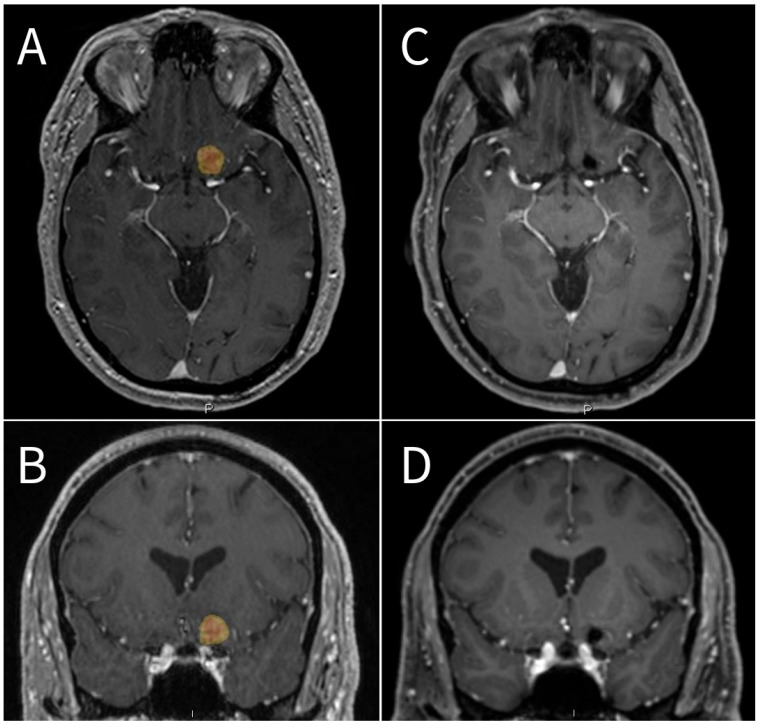
A 54-year-old male patient with BRAF V600 mutation positive melanoma on maintenance with nivolumab was diagnosed with a new left inferior frontal lobe metastasis adjacent to the left optic nerve and measuring approximately 17 mm in size. The patient was offered hypo-fractionated Gamma Knife radiosurgery in an attempt to reduce the risk of radiation-induced optic neuropathy. (**A**) Axial and (**B**) coronal, T1-weighted, post-contrast, procedural brain MRI showing the 17 mm left inferior frontal lobe lesion in close proximity to the optic apparatus. The patient was treated with a prescription dose of 25 Gy at the 50% isodose line (yellow overlay), delivered over 5 fractions. The maximum point dose per fraction delivered to the left optic nerve was 3.5 Gy. (**C**) Axial and (**D**) coronal T1-weighted, post-contrast, 30-month follow-up brain MRI showing complete tumor response. “P” for posterior and “I” for Inferior.

In the absence of randomized data, upfront HySRS is usually employed for large brain metastases not amenable to resection [13], for lesions located in eloquent areas or adjacent to dose-limiting structures [14,15] (Figure 1), and as salvage treatment for previously irradiated recurrent lesions [16]. However, the optimal tumor diameter/volume that would benefit the most from HySRS and dose-fractionation schemes remain unclear. HySRS is usually employed for brain metastases >1–2 cm in maximum diameter. The most common HySRS regimens are 27 Gray in three fractions (i.e., 3 × 9 Gy) and 30 (5 × 6 Gy) or 35 Gy (5 × 7 Gy) in five fractions (Table 1).

In a single-institution, retrospective, comparative study, Minniti et al. reported 289 consecutive patients with 343 intact brain metastases >2 cm in diameter managed with single-fraction SRS (*n* = 151) or HySRS (*n* = 138). In the single-fraction SRS cohort, brain metastases 2–3 cm in diameter were treated with 18 Gy and lesions ≥3 cm with 15–16 Gy. In the HySRS cohort, all lesions were treated with 27 Gy in 3 fractions. The cumulative 12-month local control rates were 90% for the HySRS cohort and 77% for the single-fraction SRS cohort (*p* = 0.01). The 12-month local control rates for brain metastases ≥3 cm were 54% after single-fraction SRS and 73% after HySRS (*p* = 0.02). The 1-year cumulative incidence of radiation necrosis after single-fraction SRS was 18% and 9% after HySRS (*p* = 0.01) [13].

In a prospective phase II study, Ernst et al. reported 51 patients with 72 brain metastases treated with HySRS. Patients with ≤4 brain metastases located in eloquent areas or >3 cc were included in the study. Radiation-naive patients were treated with 35 Gy in five fractions and those who received additional WBRT with 30 Gy in five fractions. Local tumor control at 6 and 12 months was 89% and 76%, respectively. The volume of the normal brain ≥23 cc receiving >4 Gy per fraction was associated with an increased risk of adverse radiation effects (14% vs. 70%) [17].

Navarria et al. reported 102 patients treated with upfront HySRS for a single brain metastasis >2 cm in maximum diameter. Lesions 2.1–3 cm in diameter were treated with 27 Gy in 3 fractions, and those 3.1–5 cm in diameter were treated with 32 Gy in 4 fractions. Local tumor progression and radiation necrosis occurred in 5.8% and 5.8% of the patients, respectively [18].

Myrehaug et al. reported 220 patients treated with upfront five-fraction HySRS for 334 brain metastases. The 12-month cumulative incidence of local failure was 23.8%. Total dose ≥ 30 Gy was associated with improved local control (33% vs. 19%). Adverse radiation effects occurred in 15.6% of the patients and were symptomatic in 9.5% of the patients [19].

Mengue et al. retrospectively reported a single-institution series of 389 patients with 400 brain metastases managed with upfront or adjuvant HySRS. Overall local control at 12 months was 76.5%, and at 24 months it was 63.9%. On multivariate analysis, resection and brain metastasis diameter less than 2.5 cm were significantly associated with improved local control. Dose fractionation was not shown to affect local control [20].

Marcrom et al. retrospectively reported single-institution outcomes of 72 patients with 182 intact metastases that were treated using a fractionation regimen of 25 Gy or 30 Gy over 5 fractions. They reported an overall 12-month local control rate of 86%, with metastases >3 cm showing significantly higher rates of local compression compared to smaller lesions (95% vs. 61%, *p* < 0.001). They also provided outcome data for local control rates based on the fractionation scheme: tumors that were prescribed 30 Gy had a higher 12-month local control rate compared to tumors treated with 25 Gy (91% vs. 75%, *p* = 0.015). The 6-month rate of symptomatic radiation toxicity was 5% [21].

The HyTEC study reported a 1-year tumor control probability of 85% and 95% for brain metastases <2 cm in diameter treated with single-fraction SRS to a dose of 18 Gy and 24 Gy, respectively. However, a single fraction dose of 18 Gy for lesions 2–3 cm and 15 Gy for those >3 cm resulted in local control of 75% and 69%, respectively. In the same study, the 1-year tumor control of lesions >2 cm treated in 3 to 5 fractions with doses ranging from 27 to 35 Gy was 80% [5].

The FRACTIONATE trial (NCT05222620) is a phase II study currently recruiting patients with an intact brain metastasis 2–4 cm in maximum diameter, randomized to receive either single-fraction SRS or HySRS. When concluded, this study will provide evidence on which approach is superior for intact, large brain metastases in terms of local control and radiation toxicity [22].

### 2.3. Adjuvant Hypo-Fractionated SRS for Resected Brain Metastases

The landmark study published in 1998 by Patchell et al. randomized 95 patients with a single, completely resected brain metastasis to observation (*n* = 46) or adjuvant WBRT (*n* = 49) starting within 28 days after surgery. WBRT resulted in a significantly reduced risk of neurologic death and brain recurrence, establishing adjuvant WBRT as the standard of care for patients with resected brain metastasis [23]. Despite improved intracranial tumor control, adjuvant WBRT does not improve survival and is associated with neurocognitive decline impacting quality of life. Consequently, due to the decreased risk of neurotoxicity and high local tumor control rates, SRS was evaluated in randomized studies as an alternative.

In 2017, a phase 3 study conducted at the University of Texas MD Anderson Cancer Center reported 128 patients with 1–3 completely resected metastases randomized (1:1) to receive single-fraction SRS within 30 days of resection or observation. Patients with Karnofsky Performance Scale score ≥70 and surgical cavity ≤4 cm were included. The radiosurgical target was defined as the surgical cavity with the addition of a 1 mm circumferential margin. Prescription doses were based on SRS target volume, with targets ≤10 cc, 10.1–15 cc, and >15 cc receiving 16, 14, and 12 Gy, respectively. Adjuvant SRS resulted in superior 12-month freedom of local recurrence rates than observation (72% vs. 43%). Patients with brain metastases ≤2.5 cm, >2.5–3.5 cm, and >3.5 cm in maximal diameter prior to resection had local tumor-free recurrence rates of >90%, 46%, and 43%, respectively. The authors suggested that dose escalation or hypo-fractionated SRS may improve local control of larger brain metastases [24].

In a multi-center, randomized-controlled, phase III trial, Brown et al. reported 194 patients assigned to receive post-surgical cavity, single-fraction SRS (*n* = 98) or WBRT (*n* = 96). Adult patients with an Eastern Cooperative Oncology Group performance status of 0–2, up to 3 unresected brain metastases, and a surgical cavity <5 cm in maximum diameter were included in the study. The radiosurgical target included the surgical cavity with a 2 mm margin and prescription dose ranging from 12 to 20 Gy according to the treated volume. Cognitive deterioration-free survival, QOL preservation, and functional independence were better in the SRS compared to the WBRT cohort [25]. Though the initial report suggested that SRS was associated with higher local failure rates than WBRT, central review showed that LF rates were not significantly higher with adjuvant SRS. Surgical cavities >3 cm and larger target volumes were associated with an increased risk of local failure [26].

These studies suggested that while single-fraction SRS is an effective treatment strategy for small-volume post-operative cavities, dose escalation is necessary to achieve satisfactory tumor control in patients with large volume cavities. Hypo-fractionated stereotactic radiosurgery to the tumor resection bed may be an alternative to single-fraction SRS to improve local control and minimize radiotoxicity (Table 2).

In a single-center retrospective study, Minniti et al. reported 101 patients treated with post-operative HySRS after complete resection of a single brain metastasis. The resection cavities were >3 cm in diameter. The radiosurgical target included the surgical cavity with the addition of a 2 mm margin. All patients were treated with a dose of 27 Gy in 3 fractions. The one- and two-year local control rates were 93% and 84%, respectively. Radionecrosis occurred in 9 patients and was symptomatic in 5 patients. The one-year risk of radionecrosis was 7%, and the two-year risk was 16%. The volume of normal brain tissue receiving 24 Gy (V24) ≥ 16.8 cm^3^ was associated with a 16% risk of radionecrosis compared to 2% for V24 < 16.8 cm^3^ [27]. The volume of normal brain tissue receiving 24 Gy (V24) ≥ 16.8 cm^3^ was associated with a 16% risk of radionecrosis compared to 2% for V24 < 16.8 cm^3^ [27].

Keller et al. reported 181 radiation naive patients treated with HySRS for 189 resection cavities. The PTV included the surgical cavity with a 2 mm margin expansion. A dose of 33 Gy was delivered in 3 fractions every 2nd day. Local control rates at 12 and 24 months were 88.2% and 86.5%, respectively. PTV > 24 mL was associated with a 3-fold increased risk of local recurrence. Radiation necrosis developed in 35 patients (18.5% of the cavities) at a median time of 15 months post-HySRS and was symptomatic in 12 patients [28].

In a retrospective study, Soliman et al. reported 122 brain metastasis patients treated with HySRS for 137 resection cavities. Prescription doses ranged from 25 to 35 Gy delivered in five fractions, with 62% treated with 30 Gy in five fractions. Local control rates at 12 and 24 months were 84% and 74%, respectively. Radiation necrosis occurred in 30% of the patients and was symptomatic in 6% [29]. Similar outcomes were reported in a multi-institutional analysis of 555 brain metastasis patients treated with a median dose of 30 Gy delivered in five fractions for 581 resection cavities. Local tumor control at one, two, and three years was 84%, 75%, and 71%, respectively. Radiation toxicity grade ≥3 occurred with a rate of 8.6% [30].

ALLIANCE A071801 (NCT04114981) is a randomized phase 3 trial expected to provide evidence on the optimal management of larger resection cavities. In this study, patients with up to 3 unresected brain metastases and a completely resected brain metastasis 2 to <4 cm in maximum diameter and a resection cavity <5 cm were stratified to receive either single-fraction SRS or HySRS to the resection cavity. The primary end point is surgical bed recurrence-free survival. This trial will provide evidence and comparative analysis of local control and radiation toxicity rates of post-operative single-fraction and HySRS [22].

## 3. Discussion

HySRS is an active area of clinical research; multiple prospective and retrospective studies provide evidence that dose hypo-fractionation affords high local tumor control as well as a low risk of symptomatic radiation toxicity. This favorable risk profile appears to be consistent for both large intact lesions and post-operative surgical cavities.

The majority of available HySRS literature has used single-fraction SRS outcomes as a benchmark for comparison of local tumor control and toxicity risk. There is growing evidence, albeit from retrospective studies, that HySRS is superior to single-fraction SRS for large intact brain metastases [13,17,18,19,20]. Minniti et al. showed that HySRS, especially for lesions with diameters >3 cm, affords significantly higher local tumor control and improved radiation toxicity profile [13]. Similarly, a recent meta-analysis pooling data from 24 studies with multiple fractionation schemes showed that HySRS could lead to lower radionecrosis rates, while maintaining high short-term local control rates compared with single-fraction SRS [31]. Based on these data, Hy-SRS for intact metastases >2 cm in diameter is conditionally recommended by the American Society for Radiation Oncology (ASTRO) [32]. This guideline conditionally recommends fractionation schemes of 27 Gy in 3 fractions or 30 Gy in 5 fractions, though outcomes of fractionation schemes of 35 Gy over 5 fractions have also been reported [17,32]. Notably, the randomized FRACTIONATE trial (NCT05222620) will evaluate the superiority of HySRS compared to single-fraction SRS [20]. In the absence of randomized study outcomes and the encouraging results from the current retrospective studies, HySRS appears to be a valid alternative to single-fraction SRS for larger, intact metastases. Both fractionation schemes appear to provide similar local control and radiation toxicity rates.

Postoperative cavity SRS with limited oligometastatic brain disease in the brain is currently the treatment of choice, given high surgical bed tumor control and reduced neurocognitive decline compared to WBRT [23,24,25]. While these studies offer prospective data on single-fraction SRS for surgical cavities, there is currently a paucity of prospective data on HySRS. However, as dose escalation might be required to achieve satisfactory tumor control in patients with large volume cavities, HySRS could be a reasonable alternative to maintain local control and reduce radiation toxicity. At this moment, only retrospective study outcomes are available; all studies report high local control rates that exceed 80%, as well as a favorable toxicity profile [27,28,29]. While not directly comparable to single-fraction SRS, it appears that HySRS offers an advantageous profile, especially for larger resection cavities. Hopefully, the ALLIANCE A071801 (NCT04114981) trial will provide evidence on the optimal management of larger resection cavities. It must be noted that these studies, including the ALLIANCE trial, present variable target delineating techniques and radiation regimens. As such, and given the lack of comparative analyses among the different regimens, the standard fractionation schemes for intact brain metastases of 27 Gy over 3 fractions and 30 Gy over 5 fractions would be applicable [32].

The transition from postoperative WBRT to focal radiation treatments (single-fraction and hypo-fractionated SRS) has resulted in an increase in leptomeningeal spread [21,22,23]. This is attributed to the surgical dissemination of disease through the cerebrospinal fluid that remains untreated with focal radiation therapies, such as SRS [32]. This observation led to the utilization of preoperative SRS as a potential mitigation strategy. The benefits of this treatment paradigm could potentially include improved target delineation, as well as pretreatment of cancerous cells that would otherwise spill into the cerebrospinal fluid during surgery and would not receive radiation. Two recent meta-analyses of retrospective studies have shown high local control rates and a low incidence of leptomeningeal dissemination [33,34]. Given these encouraging data, multiple preoperative SRS clinical trials are currently underway, including at least 3 studies evaluating preoperative HySRS (NCT05267587, NCT05341739, NCT05545007).

### Limitations

While HySRS for metastatic brain disease appears to be highly efficacious and well tolerated, with a low rate of symptomatic radiation toxicity, most of the available literature still consists of nonrandomized trials. Several real-world limitations remain that inhibit the conduct of prospective and randomized trials, including low accrual rates and standardization of radiosurgical treatment regimens. The lack of randomized data will hopefully be addressed by the ongoing randomized clinical trials, which will provide evidence on the efficacy of HySRS and allow for the optimization of treatment regimens. Additionally, advanced treatments, such as single-fraction SRS and HySRS, can be limited to specialized centers, restricting availability in many geographical regions. Even when equipment is available, underserved communities still face barriers to accessing SRS for brain metastases [35]. While there is evidence that single-fraction SRS is cost-effective compared to a combination of WBRT and SRS [36], there is a paucity of data on the cost-effectiveness of HySRS at this time.

## 4. Conclusions

Compared to single-fraction SRS, hypo-fractionated SRS appears to offer superior balance of local control and radiation toxicity for large brain metastases (>2 cm) not amenable to resection, for large volume resection cavities, and for lesions located in eloquent areas or adjacent to dose-limiting structures. Though no optimal dose fractionation scheme has been established, 27 Gy in 3 fractions and 30 Gy in 5 fractions are associated with high rates of tumor control and an acceptable toxicity profile. Ongoing randomized clinical trials are expected to provide evidence to optimize HySRS in upfront and adjuvant settings.

## Figures and Tables

**Table 1:** Selected series reporting hypo-fractionated SRS for intact brain metastases.

Author/Year	SRS Platform	Number of Patients/Brain Metastases	Median Volume (cc)/ Diameter (cm)	Prescription Dose	12-Month Local Control	Symptomatic ARE	Factors Associated with Improved LC
Minniti et al. [13], 2016	LINAC	138/164	PTV 17.9	3 × 9 Gy	90%	8%	Non-melanoma histology
Ernst-Stecken et al. [17], 2006	LINAC	51/72	PTV 13	5 × 7 Gy 5 × 6 Gy if Hx of WBRT	76%	35%	NR
Navaria et al. [18], 2016	LINAC	101/101	GTV 16.3/PTV 33.7/2.9	3 × 9 Gy (50%) 4 × 8 Gy (50%)	96%	5.8%	NR
Myrehaug et al. [19], 2022	LINAC	220/334	1.9 cm	30 Gy in 5 fr. (range, 22.5–35 Gy)	76.2%	9.5%	≥30 Gy in 5 fr.
Mengue et al. [20], 2020	CyberKnife	389/400	2.3 cm	3 × 9 Gy 5 × 6 Gy 5 × 7 Gy	76.5%	5%	<2.5 cm Prior resection
Marcrom et al. [21], 2017	LINAC	72/182	2.02/1.68	5 × 6 Gy (134 BM) 5 × 5 Gy (48 BM)	86%	5%	<3 cm Dose 30 Gy

ARE, Adverse radiation effect; BM, Brain metastasis; fr., fraction; GTV, Gross tumor volume; Hx, History; LC, Local control; NR, Not reported; PTV, planning target volume; LC, Local control; WBRT, Whole-brain radiation therapy.

**Table 2:** Selected series of HySRS for resection cavities.

Author/Year	SRS Platform	Number of Patients/Cavities	Median Volume (cc)/ Diameter (cm)	Prescription Dose	12-Month LC	Symptomatic ARE	Factors Associated with Local Failure
Minniti et al. [27], 2013	LINAC	101/101	17.5 cc	3 × 9 Gy	93%	5%	None identified
Keller et al. [28], 2017	LINAC	181/189	PTV 14.15	3 × 11 Gy	88%	18.5%	High GPA, meningeal contact, PTV > 24 mL
Soliman et al. [29], 2019	LINAC	122/147		5 × 6 Gy (range 25–35 Gy in 5 fractions)	84%	6%	Melanoma and colo-rectal histology
Eitz et al. [30], 2020	NR	558/581	PTV 23.9	5 × 6 Gy	84%	8.6%	Single BM, control of primary

ARE, Adverse radiation effects; BM, Brain metastasis; GPA, graded prognostic assessment; NR, Not reported; PTV, planning target volume.
